# Programmable four-photon graph states on a silicon chip

**DOI:** 10.1038/s41467-019-11489-y

**Published:** 2019-08-06

**Authors:** Jeremy C. Adcock, Caterina Vigliar, Raffaele Santagati, Joshua W. Silverstone, Mark G. Thompson

**Affiliations:** Quantum Engineering Technology (QET) Labs, H. H. Wills Physics Laboratory & School of Computer, Electronic Engineering & Engineering Mathematics, University of Bristol, Merchant Venturers Building, Woodland Road, Bristol, BS8 1UB UK

**Keywords:** Integrated optics, Quantum optics, Quantum information, Qubits

## Abstract

Future quantum computers require a scalable architecture on a scalable technology—one that supports millions of high-performance components. Measurement-based protocols, using graph states, represent the state of the art in architectures for optical quantum computing. Silicon photonics technology offers enormous scale and proven quantum optical functionality. Here we produce and encode photonic graph states on a mass-manufactured chip, using four on-chip-generated photons. We programmably generate all types of four-photon graph state, implementing a basic measurement-based protocol, and measure high-visibility heralded interference of the chip’s four photons. We develop a model of the device and bound the dominant sources of error using Bayesian inference. The combination of measurement-based quantum computation, silicon photonics technology, and on-chip multi-pair sources will be a useful one for future scalable quantum information processing with photons.

## Abstract (web summary)

Measurement-based quantum computing is one of the most promising approaches for photon-based universal quantum computation. Here, the authors realise a universal encoder of four-photon graph states on a silicon chip, and use Bayesian inference methods to characterise the error sources.

## Introduction

Graph states are key entangled resources for quantum information processing. They are quantum states, which can be drawn as a graph, with a qubit on each vertex and pair-wise entanglement on each edge^1^. In measurement-based quantum computing, where single-qubit measurements on a graph state drive the computation forward, particular graphs enable particular computational tasks^2^. Topological quantum error correction, relying centrally on graph states, will provide essential noise tolerance to future experimental realisations^3^. Graph states also play a central role as platforms for the simulation of complex processes and dynamics^4^, and for quantum secret sharing protocols^5^. As such, graph states have featured strongly in experiment, in both optics^6–9^ and other platforms^10^. The reconfigurable generation of arbitrary graphs, never before achieved in optics, will accelerate development of many graph-based applications.

Integrated optics promises new levels of scale for optical quantum devices. It offers robustly mode-matched, miniature components, lithographically defined in a planar process. Phase stability and matched optical path lengths are guaranteed. State-of-the-art chip-scale devices now exhibit loss and error performance approaching that of bulk and fibre systems. Quantum optical functionality has been demonstrated in all major technology platforms: lithium niobate^11^, silica^12–14^ (both lithographic and laser-written), silicon nitride^15^, gallium arsenide^16^, indium phosphide^17^, and silicon^18,19^.

Silicon devices have rapidly grown in complexity in recent years, with quantum demonstrators now exceeding 500 on-chip components^20^, and classical silicon photonic devices having thousands^21,22^. Integration with CMOS electronics could push this scale further still, by miniaturising control and interconnect functionality^22^. A quantum device’s computational power is related to the quantum configuration (Hilbert) space accessible to it. In optics, this space has *m*^*n*^ dimensions, for *n* photons scattered across *m* modes. So far, the scaling up of silicon quantum photonics has mainly involved scattering one or two photons (*n* = 1 or 2) over more and more waveguides (increasing *m*) as a route to polynomially larger Hilbert spaces^20,23^. Extending chip-scale quantum optics into the multi-pair regime, increasing *n*, is a crucial step for exponential Hilbert space scaling. Only recently has on-chip heralded interference between on-chip-generated photons been demonstrated^24,28^, though visibility is limited and no quantum information has yet been encoded.

We present a silicon quantum-optical device that can generate four photons and programmably encode them into either of the two classes^25^ of four-qubit graph state entanglement—classes closed under local unitary transformations. We refer to these two classes by their best-known members: ‘star’ |*S*_4_〉, and ‘line’ |*L*_4_〉. We observe quantum interference between photons heralded from two of the on-chip photon-pair sources, characterise the stabilisers of the star and line states, and test their multipartite nonlocality. Finally, we use Bayesian inference to access key device parameters, based on the four-photon data alone.

## Results

### Device and experiment design

**Fig. 1:**
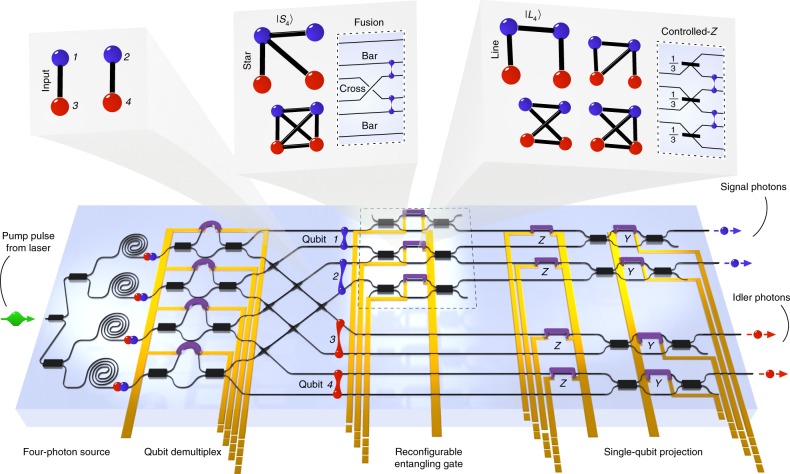
Experiment overview. A schematic of the silicon-on-insulator chip-scale device is shown, comprising: four telecommunications-band photon-pair sources, producing four photons in superposition; a qubit demultiplexer, which configures that superposition into a product of two Bell-pairs; a reconfigurable postselected entangling gate (R-PEG); and four single-qubit projection and analysis stages, formed of four Mach–Zehnder interferometers implementing qubit *Y* rotations, preceded by four *Z* rotations. An optical micrograph of the device can be found in Supplementary Fig. 1. Corresponding graph states are indicated above, starting with the two input Bell-pairs, and ending with either ‘star’ or ‘line’ graph states, for fusion or controlled-*Z* R-PEG configurations, respectively

Our device, shown schematically in Fig. 1, operates in four stages. (1) Four photons in two pairs are generated in superposition over four sources. (2) These are demultiplexed by wavelength and rearranged to group signal and idler photons. The resulting dual-rail, path-encoded qubit state is a product of Bell-pairs, |Φ^+^〉_1,3_ ⊗ |Φ^+^〉_2,4_ (with qubit indices in subscript). (3) The signal-photon qubits are operated upon by a reconfigurable postselected entangling gate (R-PEG). This can be programmed to perform either a fusion or controlled-*Z* operation, to generate star- or line-type entanglement^25^, respectively, with postselected probability 1/2 or 1/9. (*4*) We then perform arbitrary single-qubit projective measurements, using Mach–Zehnder interferometers (MZI), on the four-qubit states. A full description of the state evolution can be found in Supplementary Note 3 and Supplementary Fig. 5.

The *χ*^(3)^ process, spontaneous four-wave mixing, converts bright telecommunications-band pump pulses into quantum-correlated signal and idler photons in the spiralled silicon waveguides of our source stage^26^. Thermo-optic phase modulators provide electronic reconfigurability throughout the device. Focussing vertical grating couplers connect on-chip waveguides to optical fibre. Finally, signal and idler photons are tightly filtered in fibre (pump:photon filtering bandwidth ratio 2:1, see Supplementary Fig. 7), and registered by superconducting nanowire single-photon detectors. See Methods for more details. Using this apparatus, we measure heralded two-photon fringes, the purity of our sources, and the stabilisers of our programmed graph states with four photons.

### Heralded Hong-Ou-Mandel interference

**Fig. 2:**
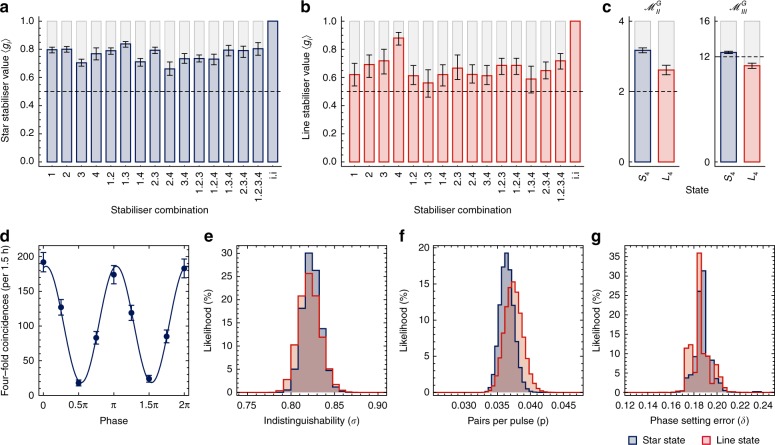
Summary of experimental data. **a**, **b** Stabiliser observables of the star and line graph states, 〈*S*_4_|*g*_*i*_|*S*_4_〉 and 〈*L*_4_|*g*_*i*_|*L*_4_〉, used to estimate state fidelity. Dashed lines indicate the *F* > 1/2 threshold to witness genuine multipartite entanglement, where *F* = mean{〈*g*_*i*_〉}. The final bar of each plot, *g*_*i*_*g*_*i*_, is the identity for any *i* ∈ {1, 2, 3, 4}. **c** Mermin parameters ${\mathscr{M}}_{II}^G$ and ${\mathscr{M}}_{III}^G$ for the |*S*_4_〉 and |*L*_4_〉 states, estimated from stabilisers measurements. Local hidden variable bounds are indicated with a dashed line. Values are reported in Table 1. **d** On-chip Hong-Ou-Mandel interference, with HOM fringe visibility of *V* = 0.82 ± 0.02. Probability distributions for the **e** indistinguishability, **f** source brightness, and **g** phase error, derived via a Bayesian parameter estimation method. All error bars represent the standard error of the mean, obtained from Monte Carlo simulations assuming a Poissonian distribution of the measured counts

Indistinguishable photons are key for high-fidelity operation. Hong-Ou-Mandel (HOM) interference, whereby two photons launched into the two ports of a beamsplitter bunch at the outputs, directly indicates their distinguishability—over all degrees of freedom—via the residual rate of antibunching from the beamsplitter outputs. When the interfering photons are heralded from entangled pairs (four photons total), non-unit photon purity also contributes to their distinguishability. On-chip path lengths are naturally matched, so rather than using the conventional time-delay HOM dip, we measure an on-chip heralded fringe^24,27^ (Supplementary Note 2 and Supplementary Fig. 4 relate these two measurements). In both measurements, the residual antibunching rate indicates the photons’ overall distinguishability. We interfere signal photons from sources 2 and 3, heralding on the two corresponding idler photons. By tuning the central R-PEG Mach–Zehnder’s phase, *ϕ*, we sweep its effective reflectivity, *R*(*ϕ*), from *R*(0) = 0, through *R*(*π*) = 1, to *R*(2*π*) = 0. At *R*(*mπ*), there is no interference, while at *R*(*mπ* + *π/*2) = 1/2, the Hong-Ou-Mandel effect occurs $(m \in {\Bbb Z})$. The measured fringe, shown in Fig. 2d, exhibits a visibility of *V* = 0.82 ± 0.02, in line with other measurements on chip^24,28^. Here, *V* = (*N*_max _− *N*_min_)/(*N*_max_  + *N*_min_), and *N*_max_ and *N*_min_ are the maximum and minimum values of the fitted sinusoid; classical light is limited to *V* < 1/3. The conventional HOM-dip-equivalent visibility, upper-bounded by the heralded purity of our photons, is *V*_HOM_ = (*N*_max _− 2*N*_min_)/*N*_max _= 0.80 ± 0.02 (see Supplementary Note 2). The photon-pair generation probability here is *p* = 0.06. We corroborate *V*_HOM_ by measuring the unheralded second-order correlation function *g*^(2)^(0) for the eight modes of our four on-chip sources, implying^29^ heralded purities between 0.82 and 0.92 (Supplementary Note 1 and Supplementary Fig. 2 contain a full listing). New on-chip parametric source designs will improve brightness and purity further^14,30,31^.

### Graph state measurements

We verify the generation of the four-photon star and line graph states (|*S*_4_〉 and |*L*_4_〉) by measuring their 16 stabilisers^32^, *g*_{*i*}_, where {*i*} is the set of generators whose product composes each stabiliser (e.g., *g*_12_ = *g*_1_*g*_2_). The four stabiliser generators of the state |*S*_4_〉 are:

1 $$g_1 = XIIZ,\ \ g_2 = IXIZ,\ \ g_3 = IIXZ,\ \ g_4 = ZZZX,$$

where *X*, *Y*, and *Z* are Pauli matrices and *I* is the identity matrix; tensor products are implied. For |*L*_4_〉, the stabiliser generators are:

2 $$g_1 = XZZI,\ \ g_2 = ZXIZ,\ \ g_3 = ZIXI,\ \ g_4 = IZIX.$$

Measurements for each stabiliser are plotted in Fig. 2a, b, for |*S*_4_〉 and |*L*_4_〉, respectively. From these, we compute fidelities, shown in Table 1, and find that both states robustly satisfy the *F* > 1/2 threshold to witness genuine multipartite entanglement^32^. These fidelities compare favourably with the first bulk-optics measurements on these states^6,33^. In these and subsequent four-photon measurements, we reduce the photon-pair generation probability to *p* = 0.03 to suppress multiphoton noise.

**Table 1:** Summary of measured parameters for on-chip graph states

State	Fidelity	${\mathscr{M}}_{\boldsymbol{II}}^{\boldsymbol{G}}$ **(2,4)**	${\mathscr{{M}}}_{\boldsymbol{III}}^{\boldsymbol{G}}$	Count rate	Counts
|*S*_4_〉	0.78 ± 0.01	3.17 ± 0.07	12.45 ± 0.13 (12, 16)	5.7 mHz	2640
|*L*_4_〉	0.68 ± 0.02	2.61 ± 0.13	10.93 ± 0.29 (12, 16)	1.1 mHz	1085
|*S*_4_〉_1,3,4_	0.77 ± 0.01	2.79 ± 0.09	6.16 ± 0.11 (6, 8)	3.3 mHz	1142
|*S*_4_〉_1,4_	0.83 ± 0.02	–	3.32 ± 0.09 (2, 4)	4.0 mHz	416
|*S*_4_〉_3,4_	0.83 ± 0.02	–	3.31 ± 0.09 (2, 4)	4.1 mHz	369
|Φ^+^〉_1,3_	0.97 ± 0.01	2.79 ± 0.01^a^	3.90 ± 0.03 (2, 4)	1.8 kHz	38003
|Φ^+^〉_2,4_	0.97 ± 0.01	2.71 ± 0.01^a^	3.88 ± 0.03 (2, 4)	1.9 kHz	41769

State fidelities, Mermin test parameters, and photon statistics are listed. Classical and quantum bounds are listed in parentheses, where they apply

^a^Indicates a Bell-CHSH test. All error bars represent the standard error of the mean, obtained from Monte Carlo simulations assuming a Poissonian distribution of the measured counts

We perform a basic measurement-based protocol^34^ by projecting various qubits of |*S*_4_〉 onto |0〉, and measuring the remaining two- and three-qubit graph states. We denote these states $\left| {S_4} \right\rangle _J \, = ( \otimes _{j \notin J}\langle 0|_j)|S_4\rangle$, where *J* is the set of remaining (un-projected) qubits. The three-qubit state |*S*_4_〉_1,3,4_ and the two-qubit states |*S*_4_〉_1,4_ and |*S*_4_〉_3,4_ can be produced by projecting qubits {2}, {2, 3}, and {1, 2} onto |0〉, respectively. Measured fidelity data for these states, along with those for the two input Bell-pairs, |Φ^+^〉_1,3_ and |Φ^+^〉_2,4_, are listed in Table 1. Notice that the two photons encoding |*S*_4_〉_1,4_ are orthogonal in colour, and have never interacted.

### Tests of multipartite nonlocality

Mermin tests let us verify the nonlocality of multipartite states^35,36^. We construct tests^32^ comprising two and three measurement settings per qubit, ${\mathscr{M}}_{II}^G$ and ${\mathscr{M}}_{III}^G$, based on the stabiliser observables of each graph state *G*. For convenience, we use ${\mathscr{M}}_{II}^G$ and ${\mathscr{M}}_{III}^G$ to indicate both the test’s operator and the modulus of its expectation value (e.g., $|\langle {\mathscr{M}}_{II}^G\rangle |$). Results are listed in Table 1 and plotted in Fig. 2c. ${\mathscr{M}}_{II}^G$ allows a choice, one for each graph symmetry, of stabilisers; we report only the optimal choice here, though all choices exceed the classical bound. Other measurement results are reported in Supplementary Table 1. We find that |*S*_4_〉 exceeds both ${\mathscr{M}}_{II}^G \, < \, 2$ and ${\mathscr{M}}_{III}^G \, < \, 12$ classical bounds. |*L*_4_〉 exceeds the classical bound for ${\mathscr{M}}_{II}^G$, but not for the more strict ${\mathscr{M}}_{III}^G$. The higher postselection penalty of the controlled-*Z*, required to generate |*L*_4_〉, results in a decreased fidelity, of which ${\mathscr{M}}_{III}^G$ is a simple rescaling.

### Understanding device performance

As quantum devices increase in complexity, the scaling of errors is of critical importance. The first step to correcting any error is to understand its source. Error models differ substantially between platforms, even within optics. Here, we develop methods for quantifying low-level performance parameters and apply these methods to our device. We seek to understand the effects of photon distinguishability, multiphoton noise, and thermo-optic phase error. Each effect is modelled independently. Since all effects contribute to the data, our estimates for each parameter are pessimistic. We apply Bayesian parameter estimation to learn the likeliest model parameters based on the four-photon stabiliser data^37^. The indistinguishability (*σ*), multiphoton emission (*p*), and random phase error (*δ*), are estimated with no prior assumptions. The resulting probability distributions of the three parameters are reported in Fig. 2e–g, for both |*S*_4_〉 and |*L*_4_〉. Fitting each with a normal distribution, we compute parameter estimates and standard deviations: *σ*_S,L _= {0.82 ± 0.01, 0.82 ± 0.01}, *p*_S,L _= {0.036 ± 0.009, 0.037 ± 0.012}, and *δ*_S,L _= {0.185 ± 0.007 rad, 0.182 ± 0.009 rad}. Our other measurements (HOM interference, *g*^(2)^, source brightness, and cross-talk—see Methods) are compatible with these estimates; the distributions for the two states, |*S*_4_〉 and |*L*_4_〉, also broadly agree. This approach can reveal additional device performance information from existing data—no new measurements are required.

To completely describe device performance a holistic error model—one that simultaneously captures all the effects—is needed. To formulate such a model requires knowledge of difficult-to-access quantities and significant computational power. A distinguishability model, for example, must have the Schmidt spectrum of each source—inaccessible from simple HOM dips—and a common basis for them. Computationally, modelling variable, high photon-number states in high-dimensional spaces is a challenge. Moreover, the three effects we studied affected the observables in a similar way and depended on the state: a holistic model may not help to effectively distinguish these effects, but tailored or adaptive measurements may help.

## Discussion

High-fidelity is crucial for many quantum information processing applications. We demonstrate entangled resources of sufficient fidelity to violate several Mermin inequalities, though future scaling will need higher fidelity still. Improved throughput (lower loss) increases fidelity: directly, multiphoton noise scales with loss, due to an increase in the relative likelihood of a multiphoton term being detected; and indirectly, shorter integration times, via higher throughput and increased rates, yield improved stability and so reduced variation in system parameters (phase setting error, calibration, pump, fibre-chip coupling, detector efficiency, etc.). Using state-of-the-art silicon photonics and customised fabrication processes, four-fold coincidence rates could be propelled to the 100-kHz regime (see Supplementary Note 4). Finally, it should be noted that our observation of rates in the 1-mHz range are comparable to rates observed in first experiments achieving historic increases in photon number (e.g., ref. ^38^).

We have demonstrated a multiphoton, multiqubit capability using standard, commercially available silicon photonic components. The techniques we have demonstrated—combining multiple postselected Bell-pairs in reconfigurable gates—can be applied to construct sophisticated chip-scale graph state generators. Although four is the largest number of dual-rail qubits for which all entanglement classes can be postselected, six- and eight-qubit devices can still access most classes: 10/11 and 73/101, respectively^25^.

Although our postselection-reliant approach to sourcing photons and preparing entanglement is not scalable, scalable approaches (e.g., those using feedforward^39–41^) must overcome many of the same challenges. We can now bring the reconfigurability and control of integrated photonics to bear on the exploration of multiphoton space. The combination of multiple photons and high-dimensional techniques^20^ will soon make vast Hilbert spaces accessible. Ultimately, postselection lets us test the components and techniques key to unlocking the huge graph states needed for photonic quantum computation^41,42^.

Graph states are, and will continue to be, a building block of large-scale quantum technology. We have demonstrated a photonic generator of arbitrary graph states, in a miniature, high-performance technology. We have encoded quantum information in more than one pair of photons generated on a chip. Future increases in photon number depend principally on improving rates, by engineering photon throughput, and dispensing with postselection. This prototype represents the next step towards a future of large-scale quantum photonic devices.

## Methods

### Experimental set-up

Pump pulses at 1544.40 nm (1.1 ps pulse duration, 500 MHz repetition rate) from an erbium-doped fibre laser (Pritel) are filtered with square-shaped, 1.4-nm-bandwidth filters and injected into the device. The average launched pump power is 4.5 mW. The pump pulse spectrum and autocorrelation are shown in Supplementary Fig. 3. The sech^2^ pulse duration is 4.80 ± 0.03 ps. Signal and idler photons are collected at pump-detuned ±4.8 nm, and filtered with square-shaped, 0.7-nm-bandwidth filters (Opneti DWDM) for spectral shaping and pump light rejection. They are detected off-chip by four superconducting nanowire single-photon detectors with 80 ± 5% efficiency (Photon Spot), operating around 0.85 K. Time-tags are generated (UQD-Logic) and converted to coincidences by bespoke software. The device is mounted using thermal epoxy and wire-bonded to an FR4 printed circuit board; temperature is stabilised using a closed-loop thermo-electric cooler. Optical coupling to fibre is via a fibre V-groove array (OZ Optics) and a 6-axis piezo-electric actuator (Thorlabs). Analogue voltage drivers (Qontrol Systems) are used to drive the on-chip phase shifters, with 16-bit and 300-μV resolution. The device was fabricated by the A*STAR Institute of Microelectronics, Singapore. A 220-nm device layer performs waveguiding, atop a 2-m buried oxide (silicon-on-insulator) with an oxide top cladding. It has an area of 1.4 × 3 mm^2^ with 500-nm-wide waveguides. Kilohertz-bandwidth thermo-optic phase modulators are formed by TiN heaters, 180 × 2 μm^2^, positioned 2 μm above the waveguide layer. See Supplementary Fig. 1 for a schematic of the experimental set-up.

### Phase shifter calibration and cross-talk

We calibrate the device’s thermo-optic phase shifters by illuminating their enclosing MZIs with a continuous-wave laser at the relevant wavelength, and applying a range of voltages to produce a fringe at the MZI output. We fit this fringe with a function *A* sin(*f* ⋅ *P*(*V*) + *ϕ*_0_) + *c*, where *P*(*V*) = *I*(*V*) ⋅ *V* is the Joule heating of the phase shifter, to find *A*, *f*, *ϕ*_0_, and *c*. By measuring the current-voltage relationship of the phase shifters and fitting them to *I*(*V*) = *ρ*_1_*V* + *ρ*_2_*V*^2 ^+  *ρ*_3_*V*^3^, we can ‘dial in’ a phase *ϕ*_d_ by numerically solving the quartic equation *ϕ*_d_ = *f* ⋅ *I*(*V*) ⋅ *V* + *ϕ*_c_. Loss-matched, evanescently coupled waveguide taps with 2% transmission are strategically placed around the device to allow independent calibration of each on-chip phase shifter.

We measure the phase deviation within one on-chip demultiplexer per unit power dissipated in the other thermo-optic modulators. A thermal cross-talk coefficient of 0.003 rad mW^−1^ results. The average power dissipated over all chip configurations used in the stabiliser measurements was 443 and 472 mW for the star and line states, respectively. These distributions indicate an average deviation from the mean of 39 and 22 mW for the two states. Working backwards, we estimate the average thermo-optic phase error is 0.12 rad and 0.065 rad, respectively. Power histograms and cross-talk fringes are shown in Supplementary Fig. 6.

### Loss

The device insertion loss is 26.1 dB for the light path through source 1 to the |0〉 output of qubit 1, after optimising the relevant phase settings. We estimate losses, based on measurements on test structures on the same die, as: 4 dB per vertical grating coupler, 0.65 dB per 2 × 2 multimode interferometer (MMI), 3 dB cm^−1^ of straight waveguide propagation, and 7.5 dB cm^−1^ of spiral waveguide propagation. All measurements are at 1544.4 nm. By including off-chip losses (3 dB), input coupling (one grating, two MMIs), and one half of the source length, we estimate that signal photons experience a loss of 19.3 dB.

### HOM-fringe visibilities

In an ideal HOM fringe the maximum is twice the background ‘distinguishable’ level of an ideal HOM dip. To calculate the equivalent dip visibility *V*_HOM_ from the maximum and minimum values measured in a fringe, we use *V*_HOM_ = (*N*_max_/2 − *N*_min_)/(*N*_max_/2) = (*N*_max _− 2*N*_min_)/*N*_max_. More details are in Supplementary Note 2.

### Measuring state fidelities

We wish to find the fidelity of our experimental state *ρ*_ex_, with a graph state *ρ*, with stabilisers {*g*_*i*_}. Since *ρ* is a stabiliser state, $\rho = \frac{1}{{2^n}}\mathop {\sum}\nolimits_i^{2^n} {g_i}$. Hence, $F = {\mathrm{tr}}[\rho _{{\mathrm{ex}}}\rho ] = \frac{1}{{2^n}}\mathop {\sum}\nolimits_i^{2^n} {{\mathrm{tr}}} [g_i\rho _{{\mathrm{ex}}}] = \frac{1}{{2^n}}\mathop {\sum}\nolimits_i^{2^n} {\langle g_i\rangle }$ (see ref. ^32^). This measurement method is used for all reported state fidelities.

Local Pauli expectation values are measured by projecting each of the 2^*n*^ eigenvectors onto each qubit’s single output waveguide and counting *n*-fold coincidences (in our experiment, *n* = 4). Summing the results of each projective measurement (total counts *C*_*j*_) by eigenvalue and normalising gives $\langle g_i\rangle = \mathop {\sum}\nolimits_j^{2^n} {\lambda _j} C_j/\mathop {\sum}\nolimits_j^{2^n} {C_j}$. Here the eigenvalue of stabiliser projector *j* is a product of its local components $\lambda _j = \mathop {\prod}\nolimits_k^n \mu _j^{(k)}$, with $\mu _j^{(k)} \in \{ - 1,1\}$ being the eigenvalue of the local operator on qubit *k*. Supplementary Note 5 contains a complete list of each state’s stabilisers.

### Mermin tests

For both |*S*_4_〉 and |*L*_4_〉, we measure every two-setting Mermin test that can be composed from its stabilisers. The tests for the star state are as follows (graph symmetries are indicated by an arrow): ${\mathscr{M}}_{II}^S = g_4(1 + g_2g_3 + g_2g_1 + g_3g_1),g_4 \to g_4g_1$ and ${\mathscr{M}}_{II\prime }^S = g_4(1 + g_i)(1 + g_j),g_4 \to g_4g_k,$ where *g*_*i*_ are the stabiliser generators and *i*, *j*, *k* = {1, 2, 3}. For the line state: ${\mathscr{M}}_{II}^L = g_1(1 + g_2)(1 + g_3),$ with *g*_2_ → *g*_2_*g*_4_ and ${\mathscr{M}}_{II{\prime}}^L = g_1(1 + g_3)(g_2 + g_4),$ with *g*_2_ → *g*_2_*g*_4_, and *g*_*i*_ → *g*_*i*_*g*_*i*+1_, for *i* ∈ {1, 2, 3, 4}. Local-realistic (‘classical’) theories obey ${\mathscr{M}}_{II}^G \, < \, 2,$ while ${\mathscr{M}}_{II}^G \, < \, 4$ for quantum mechanics.

We also report a three-setting Mermin test: ${\mathscr{M}}_{III}^G = \mathop {\sum}\nolimits_i {\langle g_i\rangle } ,$ where the sum is take over all the 2^*n*^ (16) stabilisers of the graph state. Local-realistic theories obey ${\mathscr{M}}_{III}^G \, < \, 12,$ while ${\mathscr{M}}_{III}^G \, < \, 16$ for quantum mechanics.

### Bayesian parameter estimation

We use three independent models to simulate the effects of partial distinguishability, multiphoton emission, and phase error (see Supplementary Note 6 for model details). These output a fourfold rate for each measurement setting, used to estimate a fidelity, for a range of *σ*, *p*, and *δ*. The phase error model was based on 10^4^ normally distributed Monte Carlo samples for each chip configuration, with *δ* the phase offset standard deviation. Data from each model is compared to the experimentally obtained data, and Bayesian inference learns the likeliest value for each parameter.

Consider a system described by a known model *M*(*σ*) with free parameter *σ*, a set of *N* observables ${\Pi} = \{ \pi _i\} _{i = 1}^N$ and a data set $X = \{ x_i\} _{i = 1}^N$: the general aim of Bayesian parameter estimation is to find the parameter $\bar \sigma$ that best describes the data outputted by the system. Learning $\bar \sigma$ relies on the estimation of likelihoods, over a discretised space $\{ \sigma _k\} _{k = 1}^K$ of *K* possible *σ*_*k*_: $L(\sigma _k) = \mathop {\prod}\nolimits_{i = 1}^N P(x_i|\sigma _k,\pi _i),$ where *P*(*x*_*i*_|*σ*_*k*_, *π*_*i*_) is the probability of observing *x*_*i*_ given model parameter *σ*_*k*_ and measured the observable *π*_*i*_. This probability can be calculated from the frequency of the observed data *x*_*i*_ over many samples of simulated data $\tilde x_i$. We can therefore derive the probability of *σ*_*k*_ being the parameter that best describes the data by applying Bayes’s rule:

3 $$\begin{array}{*{20}{l}} {P(\sigma _k|X,{\Pi})} \hfill & = \hfill & {\frac{{P(X|\sigma _k,{\Pi})P(\sigma _k)}}{{\mathop {\sum}\nolimits_{l = 1}^K P (X|\sigma _l,{\Pi}) \times P(\sigma _l)}}} \hfill \\ {} \hfill & = \hfill & {\frac{{\mathop {\prod}\nolimits_{i = 1}^N P (x_i|\sigma _k,\pi _i)}}{{\mathop {\sum}\nolimits_{l = 1}^K {\mathop {\prod}\nolimits_{i = 1}^N P } (x_i|\sigma _l,\pi _i)}} = \frac{{L(\sigma _k)}}{{\mathop {\sum}\nolimits_{l = 1}^K L (\sigma _l)}},} \hfill \end{array}$$

thus retrieving a probability distribution for each parameter. We have assumed the measurements to be uncorrelated and the a priori distribution of the parameters *P*(*σ*_*k*_) to be constant over the discretised range.

## Supplementary information

Supplementary Information

## Data Availability

Data and computer code that support the findings of this study are available at the University of Bristol’s data repository, *data.bris* (Digital object identifier: 10.5523/bris.2nk9fm85ssqaa2lyu4trhp5rqs). Other information is available from the authors upon reasonable request.

## References

[1] Hein M, Eisert J, Briegel HJ (2004). Multiparty entanglement in graph states. Phys. Rev. A..

[2] Hein, M., et al. Entanglement in graph states and its applications. Preprint at http://arxiv.org/abs/0602096 (2006).

[3] Raussendorf R, Harrington J, Goyal K (2007). Topological fault-tolerance in cluster state quantum computation. New J. Phys..

[4] Georgescu IM, Ashhab S, Nori F (2014). Quantum simulation. Rev. Mod. Phys..

[5] Markham D, Sanders BC (2008). Graph states for quantum secret sharing. Phys. Rev. A..

[6] Walther P (2005). Experimental one-way quantum computing. Nature.

[7] Bell B (2014). Experimental demonstration of a graph state quantum error-correction code. Nature Communications.

[8] Ciampini MA (2016). Path-polarization hyperentangled and cluster states of photons on a chip. Light.: Sci. Appl..

[9] Zhang C, Huang Y-F, Liu B-H, Li C-F, Guo G-C (2016). Experimental generation of a high-fidelity four-photon linear cluster state. Phys. Rev. A..

[10] Wang Y, Li Y, Bei Z (2018). 16-qubit IBM universal quantum computer can be fully entangled. NPJ Quantum Inf..

[11] Alibart O (2016). Quantum photonics at telecom wavelengths based on lithium niobate waveguides. J. Opt..

[12] Politi A, Cryan MJ, Rarity JG, Yu S, O’brien JL (2008). Silica-on-silicon waveguide quantum circuits. Science.

[13] Crespi A (2016). Suppression law of quantum states in a 3D photonic fast Fourier transform chip. Nat. Commun..

[14] Spring JB (2017). Chip-based array of near-identical, pure, heralded single-photon sources. Optica.

[15] Taballione, C., et al. 8 × 8 Reconfigurable quantum photonic processor based on silicon nitride waveguides. Preprint at http://arxiv.org/abs/1805.10999v2 (2018). 10.1364/OE.27.026842 31674557

[16] Dietrich CP, Fiore A, Thompson MG, Kamp M, Höfling S (2016). GaAs integrated quantum photonics: towards compact and multi-functional quantum photonic integrated circuits. Laser Photonics Rev..

[17] Sibson P (2017). Chip-based quantum key distribution. Nat. Commun..

[18] Silverstone JW, Bonneau D, O’Brien JL, Thompson MG (2016). Silicon quantum photonics. IEEE J. Sel. Top. Quant. Elect..

[19] Silverstone JW (2014). On-chip quantum interference between silicon photon-pair sources. Nat. Photonics.

[20] Wang Jianwei, Paesani Stefano, Ding Yunhong, Santagati Raffaele, Skrzypczyk Paul, Salavrakos Alexia, Tura Jordi, Augusiak Remigiusz, Mančinska Laura, Bacco Davide, Bonneau Damien, Silverstone Joshua W., Gong Qihuang, Acín Antonio, Rottwitt Karsten, Oxenløwe Leif K., O’Brien Jeremy L., Laing Anthony, Thompson Mark G. (2018). Multidimensional quantum entanglement with large-scale integrated optics. Science.

[21] Sun J, Timurdogan E, Yaacobi A, Hosseini ES, Watts MR (2013). Large-scale nanophotonic phased array. Nature.

[22] Chung S, Abediasl H, Hashemi H (2018). A monolithically integrated large-scale optical phased array in silicon-on-insulator cmos. IEEE J. Solid-State Circuits.

[23] Harris, N. C., et al. Quantum transport simulations in a programmable nanophotonic processor. *Nature Photonics* **103**, 090504–452 (2017).

[24] Faruque II, Sinclair GF, Bonneau D, Rarity JG, Thompson MG (2018). On-chip quantum interference with heralded photons from two independent micro-ring resonator sources in silicon photonics. Opt. Express.

[25] Adcock JC, Morley-Short S, Silverstone JW, Thompson MG (2018). Hard limits on the postselectability of optical graph states. Quantum Sci. Technol..

[26] Sharping JE (2006). Generation of correlated photons in nanoscale silicon waveguides. Opt. Express.

[27] Hong CK, Ou Z, Mandel L (1987). Measurement of subpicosecond time intervals between two photons by interference. Phys. Rev. Lett..

[28] Vergyris P (2016). On-chip generation of heralded photon-number states. Sci. Rep..

[29] Christ A, Laiho K, Eckstein A, Cassemiro KN, Silberhorn C (2011). Probing multimode squeezing with correlation functions. New J. Phys..

[30] Christensen JB, Koefoed JG, Rottwitt K, McKinstrie CJ (2018). Engineering spectrally unentangled photon pairs from nonlinear microring resonators by pump manipulation. Opt. Lett..

[31] Vernon Z (2017). Truly unentangled photon pairs without spectral filtering. Opt. Lett..

[32] Gühne O, Tóth G (2009). Entanglement detection. Phys. Rep..

[33] Zhao Z (2003). Experimental violation of local realism by four-photon Greenberger-Horne-Zeilinger entanglement. Phys. Rev. Lett..

[34] Raussendorf R, Briegel HJ (2001). A one-way quantum computer. Phys. Rev. Lett..

[35] Walther P, Aspelmeyer M, Resch KJ, Zeilinger A (2005). Experimental violation of a cluster state bell inequality. Phys. Rev. Lett..

[36] Ciampini MA (2017). Experimental nonlocality-based network diagnostics of multipartite entangled states. Sci. Rep..

[37] Barber, D. *Bayesian Reasoning and Machine Learning* (Cambridge University Press, Cambridge, England, 2012).

[38] Bouwmeester D (1997). Experimental quantum teleportation. Nature.

[39] Knill E, Laflamme R, Milburn GJ (2001). A scheme for efficient quantum computation with linear optics. Nature.

[40] Gimeno-Segovia M (2017). Relative multiplexing for minimising switching in linear-optical quantum computing. New J. Phys..

[41] Gimeno-Segovia M, Shadbolt P, Browne DE, Rudolph T (2015). From three-photon Greenberger-Horne-Zeilinger states to ballistic universal quantum computation. Phys. Rev. Lett..

[42] Rudolph T (2017). Why I am optimistic about the silicon-photonic route to quantum computing. APL Photonics.

