# Supplementary material for: Programmable four-photon graph states on a silicon chip
Source: Nat Commun. 2019 Aug 6;10:3528. doi: 10.1038/s41467-019-11489-y (PMC6684799; doi:10.1038/s41467-019-11489-y)
Supplement: Supplementary file 1 — Supplementary Information [file 41467_2019_11489_MOESM1_ESM.pdf]

Supplementary Information - Programmable four-photon graph states on a silicon chip

Jeremy C. Adcock et al.

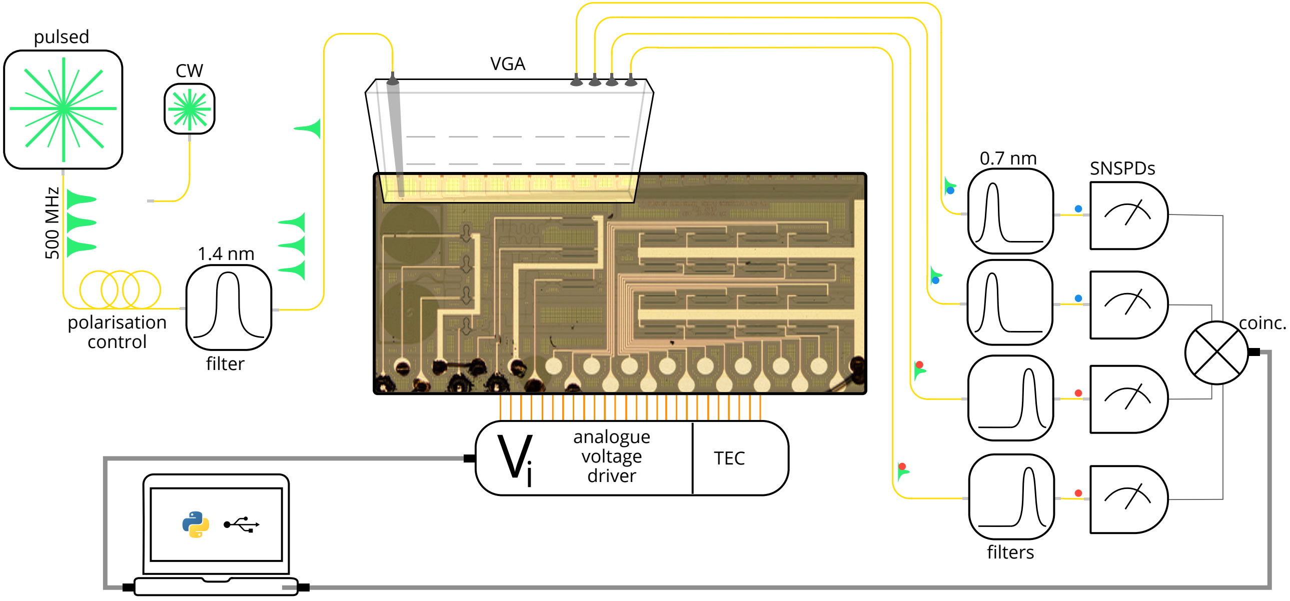

Supplementary Figure 1: Experimental set up for testing our device. Bright laser light (pulsed for single photon operation, continuous wave for calibration) at 1544 nm is input to our device via polarisation control and a 1.4 nm filter. Optical access to the device is via a v-groove array fibre array. Extracted single photons are sent through 0.7 nm filters at the signal and idler bands (1549 and 1539 nm respectively) and detected in superconducting nanowire single photon detectors (SNSPDs, Photon Spot). For calibration, the filters are removed and the SPSPDs replaced with bright-light photodiodes. The phase shifters of the chip are controlled by analogue voltage controllers (Qontrol Systems). Timetags are generated (UQD-Logic) and converted to coincidence output data. A micrograph of the device is shown the centre.

Supplementary Note 1 - Photon-pair source characterisation

Isolating each source using the qubit demultiplexers, we measure the rate of two-photon coincidences while changing the launched power. We find a quadratic relationship (Supplementary Figure 2), confirming that a third-order non-linear process (SWFM) is responsible for their generation. Further, we find that our Bell state generators are well balanced—a necessary condition for the production of high fidelity Bell states [1]. The relative brightness of the two Bell pair sources is not important for state fidelity, but does effect the four-photon rate.

We calculate a coupling-independent metric for the probability of each pulse to generate one or more photon pairs in source i, p_i, by solving a set of simultaneous equations [2]: $C = \eta_s\eta_i R$, $S_s = \eta_s R$, $S_i = \eta_i R$. Here, η_s, η_i are the signal and idler transmissions, and C and S_s, S_i are the coincidence and singles rates, and R is the pair generation rate. We use this to monitor mutltiphoton emission. Supplementary Figure 2 shows estimated pair production probability, p_i as a function of pump attenuation. We can also solve for the heralding efficiency of each photon. Taking the first value from Supplementary Figure 2a, we measure the transmission for the signal photons to be -20.3 dB, -20.7 dB, -19.1 dB and -18.8 dB. For the idlers we find -22.2 dB, -23.1 dB, -22.3 dB and -22.0 dB.

We measure the unheralded $g^2(0)$ of both photons from our four on-chip sources to estimate their spectral purity [3]. The measured purities, all of which are between 0.82 and 0.92, are shown in Supplementary Figure 2. Here, spectral entanglement of the photon pairs is minimised by tight off-chip filters (Supplementary Figure 7). The launched power for this measurement was calibrated such that $p_i = 0.03$.

Supplementary Note 2 - Hong-Ou-Mandel interference

Measuring quantum interference in a Hong-Ou-Mandel (HOM) dip is the canonical way to establish two particles' indistinguishability. A conventional HOM-dip experiment launches two single photons on a beamsplitter and observes the rate of residual antibunching at two output ports. Perfectly indistinguishable photons always bunch at the output, while distinguishable ones bunch with 50% probability. By continuously tuning some parameter that distinguishes the photons (often the time of arrival of one of the photons), the coincidences 'dip' where the particles are most indistinguishable due to quantum interference. This dip has visibility defined $V_{\mathrm{HOM}} = (a-b)/a$ (for a reference level a and dip minima b, see Supplementary

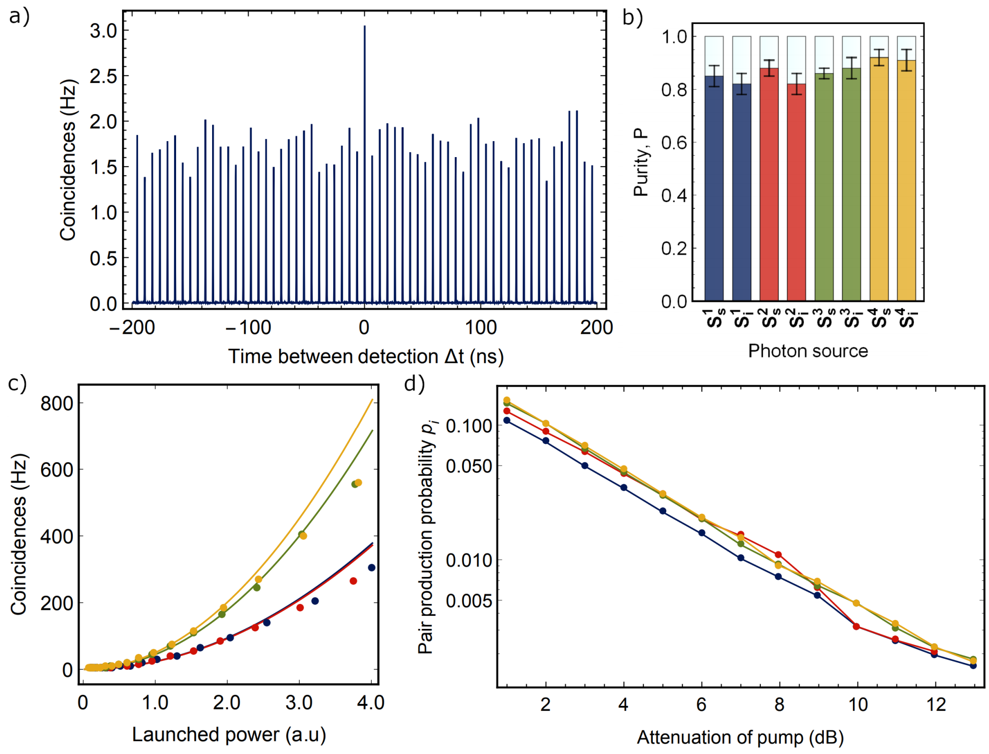

Supplementary Figure 2: Characterising on-chip photon-pair sources. a) a typical histogram measuring an unheralded $g^{(2)}(0)$, which is the ratio between the area under the central and side peaks. This histogram was measured of signal photons from source 1, for $p_1 = 0.03$. The purity[3] is $g^{(2)}(0)-1$. b) purities of the signal and idler photons of each source measured in this way. Error bars are 1 standard deviation. c) pair generation rates and launched power for the four on-chip photon pair sources. Here, the fits take into account only the first 13 points, before other nonlinear loss becomes significant. d) effect of pump attenuation on the probability to generate at least one photon pair. All error bars represent the standard error of the mean, obtained from Monte Carlo simulations assuming a Poissonian distribution of the measured counts.

Figure 4), which corresponds precisely to the indistinguishability of the photons).

Our nonlinear sources have partially entangled entangled joint spectra—our signal photons can be partially distinguished from one another by measurement of their partner idler photon (the heralded photons are not pure). Filtering the signal and idler photons can carve a more separable joint spectrum from one that is entangled (see Supplementary Figure 7) though this comes at the cost of rate. We find that a reasonable trade off is made by filtering our pump pulses with 1.4 nm square filters before insertion and filtering our single photons with 0.7 nm square filters after extraction (see Supplementary Figure 7)

2.1 On-chip Hong-Ou-Mandel fringe

A HOM dip experiment is challenging to implement using integrated waveguide circuits—there is no easily controllable degree of freedom to introduce distinguishability (cf. time of arrival in bulk optics). With particular reference to arrival time, integrated optics is inherently path length matched, so arrival-time variation is not necessary—the photons are always maximally indistinguishable in arrival time. Hence we perform a HOM-*fringe*[4,5]. Here, a reference rate, N_{max}, is provided by the pass-through and swap settings of an MZI ($\phi = m\pi$ for $m \in \mathbb{Z}$), while the residual antibunching rate, N_{min}, is provided by the MZI acting as a beamsplitter ($\phi = \pi/2 + m\pi$).

In a HOM dip, when the input photons are totally distinguishable, the photons scatter randomly and independently, resulting in bunching half of the time. In the HOM fringe, however, no photon bunching occurs when the MZI is set to pass-through and to swap. Knowing this, it is easy to normalise the HOM interference measurement: $a = N_{\text{max}}/2$. The interfering case, with the residual antibunching rate indicating the distinguishability, is identical between the two experiments, and so $N_{\text{min}} = b$. Hence:

$$V_{\text{HOM}} = (N_{\text{max}}/2 - N_{\text{max}})/(N_{\text{max}}/2) = (N_{\text{max}} - 2N_{\text{min}})/(N_{\text{max}}). \tag{1}$$

A pictorial comparison between the HOM dip and fringe experiments is shown in Supplementary Figure 4.

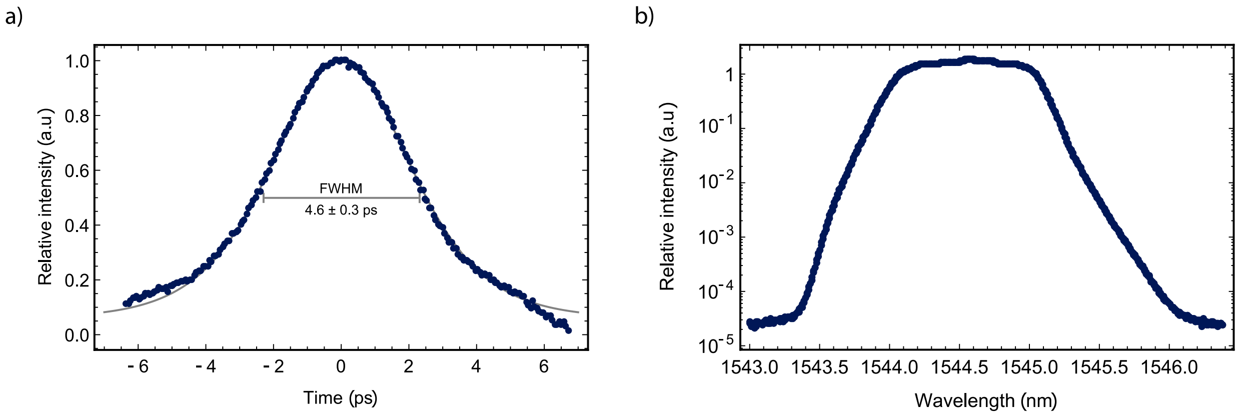

Supplementary Figure 3: Properties of our pump laser pulses after filtering. a) Pump pulse shape found by autocorrelation measurement (after filtering—see Supplementary Figure 7). A sech^2 fit, with duration 4.80 ± 0.03 ps, is shown. This implies a peak power of 1.0 W (for our average power of 4.5 mW). b) Spectrum of the fitered pump pulse (log scale).

Supplementary Note 3 - Four-qubit state evolution

After spectral demultiplexing, our device postselects the state $|\Psi\rangle = |\Phi^+\rangle_{1,3} \otimes |\Phi^+\rangle_{2,4} = \frac{1}{2}(|0000\rangle + |0101\rangle + |1010\rangle + |1111\rangle)$ (see Supplementary Figure 5). Setting the R-PEG to perform fusion we generate:

$$|\mathrm{GHZ}\rangle = \frac{1}{\sqrt{2}}(|0101\rangle + |1010\rangle). \tag{2}$$

After postselection. Then, applying a local unitary yields the star graph state:

$$H_1 X_2 H_2 H_3 X_4 |\mathrm{GHZ}\rangle = |S_4\rangle. \tag{3}$$

Similarly, setting the R-PEG to perform a CZ generates:

$$|C_4'\rangle = \frac{1}{2}(|0000\rangle - |0101\rangle + |1010\rangle + |1111\rangle). \tag{4}$$

After postselection. Then, applying a local unitary yields the star graph state:

$$Z_1 H_3 H_4 |C_4'\rangle = |S_4\rangle. \tag{5}$$

Supplementary Note 4 - Improving loss

Our device was manufactured on a commercial process using standard recipes. After generation, our single photons traverse a single grating coupler, six 2×2 beamsplitters and 3 cm of waveguide, and experience 1 dB of detector loss. State-of-the-art components boast losses of $\eta_g = 0.58$ dB per grating coupler[6], $\eta_b = 0.15$ dB per beamsplitter[7], $\alpha_{\mathrm{dB\ cm^{-1}}} = 0.34$ dB cm^{-1} propagation loss in single-mode waveguides[8], and $\eta_d = 95.5\% = -0.20$ dB detection efficiency is commercially available[9]. By using these components, the total source-to-detector loss of our device could be as low as:

$$\eta_d \eta_g \eta_b^6 10^{L\alpha_{\mathrm{dB\ cm^{-1}}}} = 0.20 \text{ dB} + 0.58 \text{ dB} + 0.15 \text{ dB} \times 6 + 0.34 \text{ dB cm}^{-1} \times 3 \text{ cm} = 2.60 \text{ dB}. \tag{6}$$

Which should be compared to the 19.3 dB experienced by photons in our current experiment. This would increase the measured four-fold coincidence rate by a factor of $4 \times (19.3 - 2.6)$ dB $= 66.9$ dB or about $5{,}000{,}000$ times: from the 10-mHz range to the 100-kHz one.

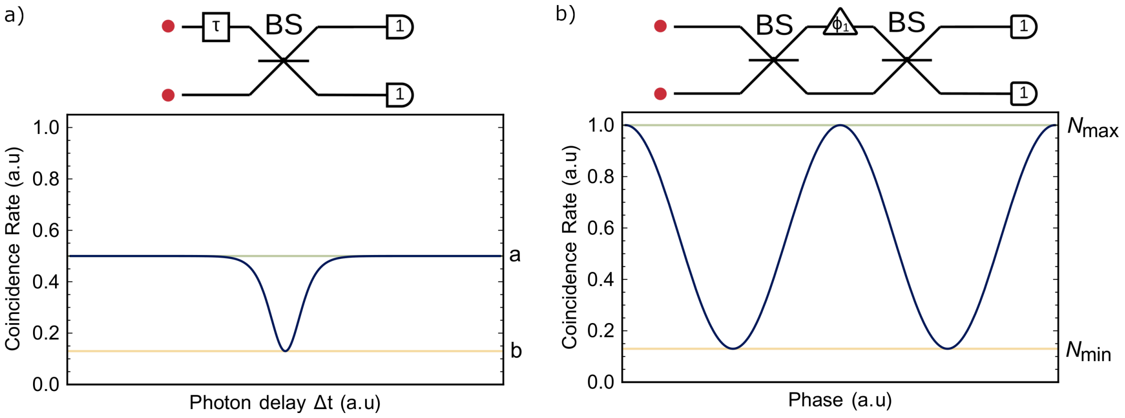

Supplementary Figure 4: Comparison of HOM interference measurements. Interferometers are shown with a cartoon of their measurement results below them. a) A degree of freedom that distinguishes two photons, here the time of arrival at a beamsplitter, is tuned. Coincidences reach a minimum when the photons are most distinguishable in the tunable degree of freedom. A reference level is provided in the distinguishable case, where the photons act independently, causing a coincidence half of the input pairs. b) a MZI acts as a completely tunable beamsplitter. The fringe has maxima at the identity and swap configurations, where every pair causes a coincidence, and minima when configured to be a 50:50 beamsplitter. The maxima of the fringe are twice the maxima (reference level) of the dip: $a = N_{\max}/2$. Hence $V_{\text{HOM}} = (a-b)/a = (N_{\max}/2 - N_{\min})/(N_{\max}/2) = (N_{\max} - 2N_{\min})/N_{\max}$.

Supplementary Note 5 - Measurement settings

5.1 Star graph stabilisers

With four qubits, there are four different star graph states. These have each qubit $i \in \{1,2,3,4\}$ as the centre of the star. We generate and measure the star where qubit four is the centre. This has stabilisers:

$$g_1 = XIIZ, \quad g_2 = IXIZ, \quad g_3 = IIXZ, \quad g_4 = ZZZX, \tag{7}$$

$$g_{12} = XXII, \quad g_{13} = XIXI, \quad g_{14} = YZZY, \quad g_{23} = IXXI, \quad g_{24} = ZYZY, \quad g_{34} = ZZYY, \tag{8}$$

$$g_{123} = XXXZ, \quad g_{124} = -YYZX, \quad g_{134} = -YZYX, \quad g_{234} = -ZYYX, \quad g_{1234} = -YYYY, \quad g_{ii} = IIII. \tag{9}$$

The expectation values of these stabilisers are used to calculate a state fidelity with the star graph state $F = \sum_i^{16} \langle S_{\text{i}} \rangle$. Conveniently, some of these expectation values can be calculated from the same data. $\langle IIII \rangle = 1$, for example, is trivial. More pertinently, $\langle XIIZ \rangle$, $\langle IXIZ \rangle$, $\langle IIXZ \rangle$, $\langle XXII \rangle$, $\langle XIXI \rangle$, $\langle IXXI \rangle$ can all be calculated from the data collected for $\langle XXXZ \rangle$—a state fidelity can be estimated by measuring coincidence rates for $9 \times 16 = 144$ projectors, rather than $16 \times 16 = 256$.

To understand this technique, note that for a qubit, $I = |a\rangle\langle a| + |b\rangle\langle b|$ for $\langle a|b\rangle = 0$. Measuring the identity can be simulated by taking any complete set of measurements and setting both eigenvalues to $+1$. For example, to calculate $\langle S' \rangle = \langle IXXI \rangle$ from the data collected for $\langle S \rangle = \langle XXXZ \rangle$, the coincident rates are summed such that the eigenvalues of the projectors on qubits 1 and 4 do not contribute to the measurement outcome—only the eigenvalues of qubits 2 and 3 contribute to the eigenvalue of S'.

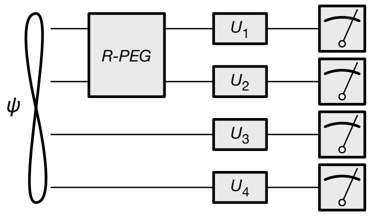

Supplementary Figure 5: Quantum circuit of the device. To generate the star, $|S_4\rangle$, we set the R-PEG to fusion and $U_1 = H$, $U_2 = XH$, $U_3 = H$, $U_4 = X$. To generate the line, $|L_4\rangle$, we set the R-PEG to Controlled-Z and $U_1 = Z$, $U_2 = I$, $U_3 = H$, $U_4 = H$.

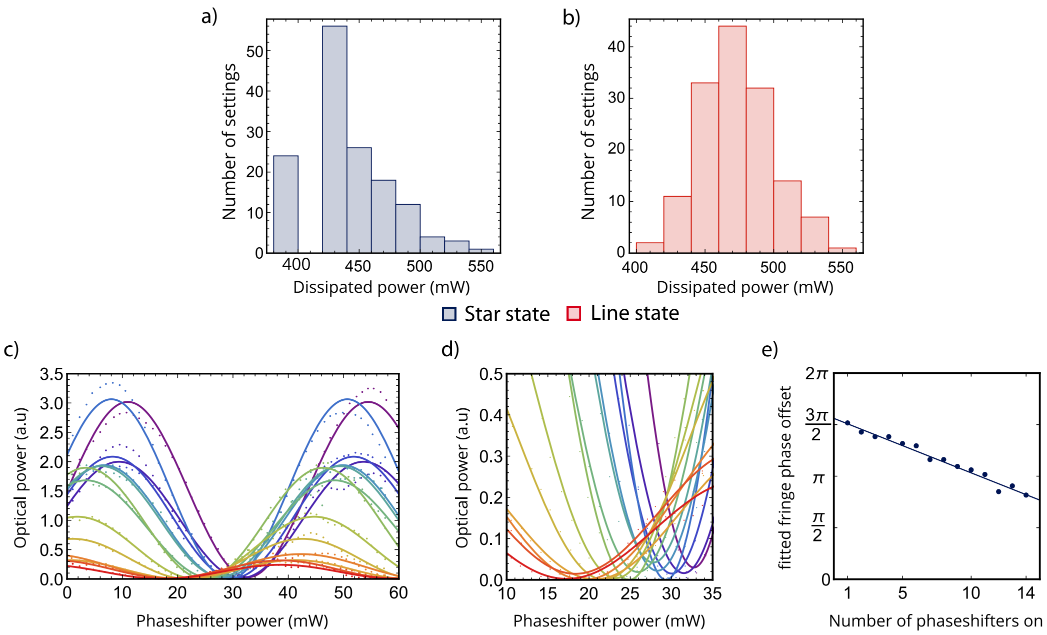

Supplementary Figure 6: Crosstalk on our device. a) Histogram of the power dissipated by the on-chip thermo-optic phase shifters for the star state. b) Histogram of the power dissipated by the on-chip thermo-optic phase shifters for the line state. c) bright light fringes from one of the on-chip qubit demultiplexers. Starting with all phase shifters switched off, they are sequentially switched on to 8 and a fringe scanned. d) detail showing the phase drift as more heat is dissipated to the chip. e) phase offsets of the fringes as phase shifters are sequentially turned on. We extract a thermal phase crosstalk coefficient of 0.003 rad/mW

5.2 Line graph stabilisers

With four qubits, there are twelve different line graph states. We generate and measure the line with edges (3,1), (1,2), (2,4), which has stabilisers:

$$g_1 = XZZI,\quad g_2 = ZXIZ,\quad g_3 = ZIXI,\quad g_4 = IZIX, \tag{10}$$

$$g_{12} = YYZZ,\quad g_{13} = YZYI,\quad g_{14} = XIZX,\quad g_{23} = IXXZ,\quad g_{24} = ZYIY,\quad g_{34} = ZZXX, \tag{11}$$

$$g_{123} = -XYYZ,\quad g_{124} = -YXZY,\quad g_{134} = YIYX,\quad g_{234} = IYXY,\quad g_{1234} = XXYY,\quad g_{ii} = IIII \tag{12}$$

As with the star, however, the number of measured stabilisers needed for a fidelity estimate can be reduced to 9. $\langle XZZX\rangle$ yields $\langle XZZI\rangle$, $\langle IZIX\rangle$, $\langle XIZX\rangle$. $\langle ZXXZ\rangle$ yields $\langle ZXIZ\rangle$, $\langle ZIXI\rangle$, $\langle IXXZ\rangle$. $\langle YZYX\rangle$ yields $\langle YZYI\rangle$, $\langle YIYX\rangle$. Finally, $\langle ZYXY\rangle$ yields $\langle ZYIY\rangle$, $\langle IYXY\rangle$.

Supplementary Note 6 - Quantum photonic simulation

We model the effect of three main sources of infidelity in our device: partial distinguishability, multiphoton emission, and phase control noise. To do this, we write the photonic state generated by our on-chip sources, propagate it through the linear optical unitary of the device, and generate 'coincidence' output data of the same type as the experiment. This is done independently for each of the three noise sources considered. The results of these simulations are used to inform our Bayesian parameter estimation for the different noise sources.

We use two simulators, POMOS and PERM, to to establish the deleterious effects in our device, and to inform our Bayesian parameter estimation. POMOS is model spectral entanglement in our photon-pair sources, while PERM is used to model multiphoton emission. Finally, PERM is also used to calculate the effect of random phase errors, using a Monte Carlo approach.

6.1 Polynomial of mode operators simulator: POMOS

In this simulator, photonic states are represented by a polynomial of photon creation operators, a, where we drop the dagger for clarity. An example of a state stored in this way is $(ac+bd)/\sqrt{2}$, representing the Fock state $(|1010\rangle + |1010\rangle)/\sqrt{2}$.

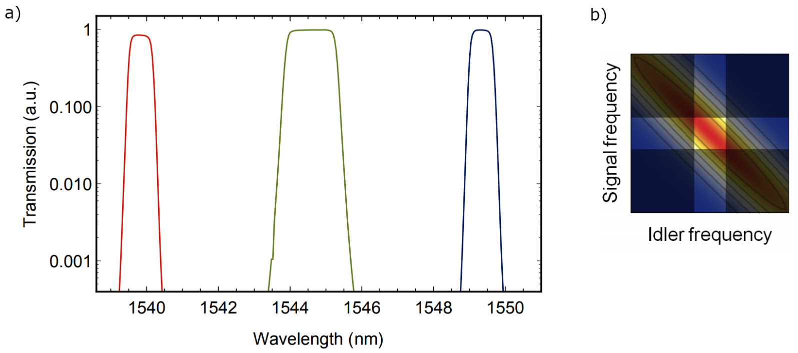

Supplementary Figure 7: Carving out pure photon pairs from spectrally entangled SFWM. a) transmission spectra of the off-chip filters. b) a cartoon of a typical joint spectral amplitude a biphoton generated via SWFM. Filters, which would remove spectral correlation, are indicated.

From here it is simple to implement unitary transformations on the state. For example, a beamsplitter between modes a and b:

$$a \to \frac{1}{\sqrt{2}}(a+ib), \tag{13}$$

$$b \to \frac{1}{\sqrt{2}}(b+ia). \tag{14}$$

The set of on-chip operations is completed by the phase shifter, which transforms a mode $a \to e^{i\phi}a$ for some phase ϕ. Concatenation of these two operations can construct any optical unitary.

Multiple frequency entangled sources of squeezed vacuum on chip

To simulate our device, we must describe the state that is generated by the on-chip sources, ψ_{in}. In a quantum optical treatment, these sources each generate a two-mode squeezed vacuum state, from which the two-photon component is postselected to yield a photon pair source. In this section we will focus on spectral entanglement in the sources, so its effect may be propagated in to meaningful output statistics by POMOS.

To summarise the notation used in this section, spatial modes are labelled by characters from the latin alphabet, frequency modes are labelled by superscript, and whether the photon is at signal or idler wavelengths is labelled by the subscript. Here the signal and idler photons are non-degenerate and so are orthogonal.

Two-mode squeezed vacuum in spatial modes a_{s} and a_{i}, with squeeze parameter $\xi^{(a)}$ has quantum state:

$$|\psi_a\rangle = \sqrt{1-|\xi^{(a)}|^2}\sum_{n=0}^{N}\frac{(-\xi^{(a)})^n(a_{\text{s}}a_{\text{i}})^n}{n!}|0\rangle. \tag{15}$$

Where $\xi^{(a)} = ie^{i\text{Arg}(\zeta^{(a)})}\tanh(\zeta^{(a)})$. This is normalised for $N \to \infty$. For small ξ, $|\psi_a\rangle$ is dominated by vacuum, with an $O(\xi)$ two-photon component. This two-photon component is our photon pair source.

In the above a_{s} and a_{i} are clearly separable, however this is not representative of most states generated by nonlinear processes. In general, energy conservation causes the photon pair to be entangled. Instead we write the state with a discrete joint spectral amplitude (JSA) $J_a(k,l)$, where k and l index discrete spectral modes. This allows us to express spectral entanglement in the biphoton (c.f. a continuous JSA $J(\omega_k,\ \omega_l)$):

$$a_{\text{s}}a_{\text{i}} \to \sum_{l=1}^{L_a}\sum_{k=1}^{K_a} J_a(k,l)a_{\text{s}}^{(k)}a_{\text{i}}^{(l)}. \tag{16}$$

Here, $\sum_{l=1}^{L_a}\sum_{k=1}^{K_a}|J_a(k,l)|^2 = 1$. Using an (orthogonal) Schmidt basis only $J_a(k,k)$ is populated, and:

$$|\psi_a\rangle = \sqrt{1-|\xi^{(a)}|^2}\sum_{n=0}^{N}\frac{1}{n!}\Big(\sum_{k=1}^{K_a} -\xi^{(a)}J_a(k,k)a_{\text{s}}^{(k)}a_{\text{i}}^{(k)}\Big)^n \quad |0\rangle. \tag{17}$$

This describes an entangled biphoton state in terms of its Schmidt decomposition. This is a tensor product of squeezed states:

$$|\psi_a\rangle = \bigotimes_{k=1}^{K_a} S(\xi_k^{(a)})|0\rangle. \tag{18}$$

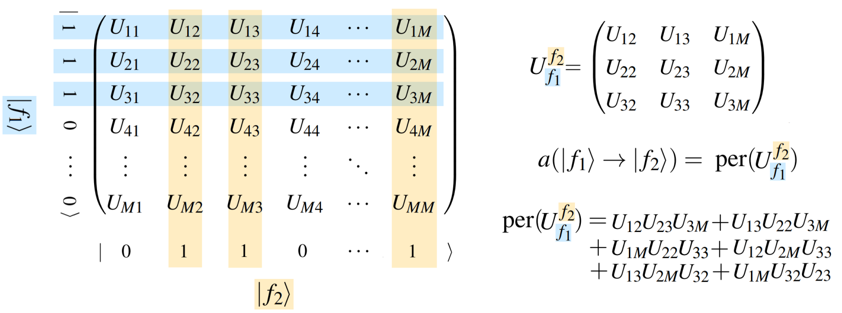

Supplementary Figure 8: Relation of the permanent to linear optical transitions. The transition amplitude to transform $|f_1\rangle$ into $|f_2\rangle$ via linear optical unitary U, $a(|f_1\rangle \to |f_2\rangle)$, is calculated by extracting a submatrix $U_{f_1}^{f_2}$ and calculating its permanent.

To model our four sources, we take the tensor product of four of these frequency entangled two-mode squeezed vacuum states:

$$|\psi_{\mathrm{in}}\rangle = |\psi_a\rangle \otimes |\psi_b\rangle \otimes |\psi_c\rangle \otimes |\psi_d\rangle. \tag{19}$$

This state can be propagated through the device unitary to generate the full output state. Since our photon detectors can not resolve frequency, we incoherently sum over the frequency degree of freedom to generate output statistics. To generate a four-fold 'rate' for each measurement setting, the probabilities of each term that contains a photon in all four output spatial modes are summed with no interference between photons of different frequency.

Controlling distinguishability in Pomos

To progress, we must set the overlaps (between different sources) and amplitudes of the ensemble of Schmidt modes $J_i(k,k)$. This is nontrivial. It is possible to do tomography on the Schmidt modes of a photon pair source[10], but this is beyond the scope of our experiment. Further, simulating an arbitrary Schmidt spectrum would require formidable computational resources.

Instead, we make an approximation: each source i has amplitude in $J_i(1,1)$, corresponding to the desired amount of indistinguishability. The rest of the state occupies some unique mode $k = 2,3,4,5$ for sources a, b, c, d respectively. In this way, each source occupies two modes, one which is shared with the other sources, and another which is completely distinguished from them. This has the advantage of limiting the size of the simulation by keeping modes completely orthogonal (or completely overlapping), while keeping the number of modes to a minimum. For low values of indistinguishability, this approximation is likely poor, but for our expected purities of $P > 0.8$, where $J(1,1)$ dominates, two unbalanced Schmidt modes is a good approximation.

6.2 Permanent computing linear optics simulator: Perm

'Multiphoton' (here $N > 2$ pairs) emission can be included in Pomos by setting N at the desired level. In practice, however, both multiphoton emission and partial distinguishability require propagating an exponential number of terms, which is infeasible using the symbolic manipulation of Pomos (at least with our implementation in Mathematica). Instead, we use linear optics' surprising relationship the matrix permanent[11].

Analogous to the determinant, the permanent is a function of each of the entries of the matrix, outputting a single (complex) number. The entries are multiplied according to permutations of the symmetric group (of size n for an $n \times n$ matrix) and then summed. In the determinant, summands whose permutation is odd have negative sign, whereas in the permanent, each summand has positive sign.

$$\mathrm{per}(M) = \begin{pmatrix} a & b & c \\ d & e & f \\ g & h & i \end{pmatrix} = aei + afh + bdi + bfg + cdh + ceg \tag{20}$$

In short, the probability of an n-photon Fock state, $|f_1\rangle$, to transition to another Fock state, $|f_2\rangle$, via a $M \times M$ linear optical unitary, U, is related to a the permanent of an $n \times n$ matrix which is selected using the occupations of $|f_1\rangle$ and $|f_2\rangle$. More precisely, to calculate the probability amplitude for a state $|f_1\rangle = |j_1, j_2, \ldots, j_M\rangle$ to transition to another $|f_2\rangle = |j'_1, j'_2, \ldots, j'_M\rangle$, we select a submatrix $U_{f_1}^{f_2}$, of U by taking j_i copies of the ith row and j'_i copies of the ith column and calculate its permanent

(see Supplementary Figure 8). The probability amplitude for a transition from $|f_1\rangle$ to $|f_2\rangle$ is:

$$a(|f_1\rangle \to |f_2\rangle) = \frac{\mathrm{per}(U_{f_1}^{f_2})}{\sqrt{j_1!\ j_2!\ \ldots\ j_M!\ j'_1!\ j'_2!\ \ldots\ j'_M!}}. \tag{21}$$

Using this method, any output statistics can be calculated, including full output state. To find the amplitude in some output Fock state $|F\rangle$, given some input state $|\psi_{\mathrm{in}}\rangle = \sum_i \alpha_i |f_i\rangle$, we sum over the transition amplitudes from each input Fock state $a_F = \sum_i \alpha_i a(|f_i\rangle \to |F\rangle)$.

The permanent is a famously hard computation problem, belonging to computational complexity class #P, and as such is generally intractable for large n. Still, our Mathematica implementation of PERM is an order of magnitude faster than the symbolic manipulation used by our implementation of POMOS.

Tuning multiphoton emission with PERM

To apply this simulator to calculate the effect of multiphoton emission on our device, we feed in our input state:

$$|\psi_{\mathrm{in}}\rangle = \left(\sqrt{1-|\xi|^2}\sum_{n=0}^{N}(-\xi)^n (a_s a_i)^n |nn\rangle\right)^{\otimes 4}, \tag{22}$$

and compute output statistics with PERM for a range of ξ. To do this, the state must be truncated at some N. We calculate the effect of multiphoton terms on HOM dip visibility for up to $N = 6$ pairs (see Supplementary Figure 9). For our estimated source brightness of $p = 0.03$, there is negligible difference for $N > 4$.

Fundamentally, our device measures the probability to detect $|10101010\rangle_f$ (only these outputs are available on the device) which changes according to the measurement setting. These rates are used to we calculate all further statistics and data. However, our single-photon detectors are not photon number resolving: they cannot distinguish if one photon was present, or if it was two, or five. To model this output, PERM incoherently sums over each Fock output that causes a four-fold coincidence. For example, our detectors cannot discriminate $|10101010\rangle_f$ from $|10102020\rangle_f$—both Fock states contribute to our statistics.

Multiphoton emission and photon loss

When combined with multiphoton emission, loss has a significant effect on the output statistics: a detector with efficiency $0 < \eta < 1$ is more likely to detect two photons than one—you can lose either of them, and still get a click. For this reason, a correction to the output statistics must be made to account for. This effect is larger the smaller η is—for our heralding efficiency of of -19 dB ≈ 0.01, the correction is vital.

Since we measure in coincidence, we are only interested in Fock states which have at least one photon in each of the four measured modes $|F\rangle = |k_1, 0, k_2, 0, k_3, 0, k_4, 0\rangle$, where k_i are the occupations of the modes we measure ($i \in \{1,2,3,4\}$). Photons in modes other than these have no effect. To calculate the probability of a four-fold coincidence, we multiply the independent probabilities of detectors $i \in \{1,2,3,4\}$ to click given k_i photons. We sum over each case where a photon was lost such that the detectors still give a coincidence. This is a binomial expansion for each of the k_i photons being lost in each source i. For $\eta_1 = \eta_2 = \eta_3 = \eta_4 = \eta$:

$$\Gamma(|F\rangle) = \sum_{l_1=0}^{k_1-1}\sum_{l_2=0}^{k_2-1}\sum_{l_3=0}^{k_3-1}\sum_{l_4=0}^{k_4-1}\binom{k_1}{l_1}\binom{k_2}{l_2}\binom{k_3}{l_3}\binom{k_4}{l_4}\eta^{\Sigma k_i - \Sigma l_i}(1-\eta)^{\Sigma l_i}. \tag{23}$$

This modifier contributes to all data simulated with PERM. For each term $|F\rangle$ in the output state with amplitude α_F the coincidence probability is $\Gamma(|f_2\rangle)|\alpha_F|^2$.

6.3 Bayesian parameter estimation

To learn the most likely noise parameters of our measured data with Bayesian parameter estimation, we generate three sets of data using PERM and POMOS—one for each noise parameter. PERM was used to generate output statistics for our device for a range of source brightness $p \approx |\xi|^2$. Similarly, POMOS was used to generate output statistics for a range of indistinguishabilities σ (according to the model of Supplementary Note 6.1). To estimate the phase noise, PERM was used to model $N = 2$ pairs of photons on the device, with each phase shifter comprising the device unitary subject to Gaussian noise with width δ. 10^4 Monte Carlo samples were averaged for each δ to provide a mean output statistic. Supplementary Figure 10 shows output data for two stabilisers for the line and star graph states. Simulated data shows the effect of each noise source. The simulated data is for the parameter that the Bayesian method found best explained the measured data. Supplementary Figure 9 shows the how the fidelity of the two states scales with the parameters, as well as the effect of loss and the choice of N on multiphoton noise.

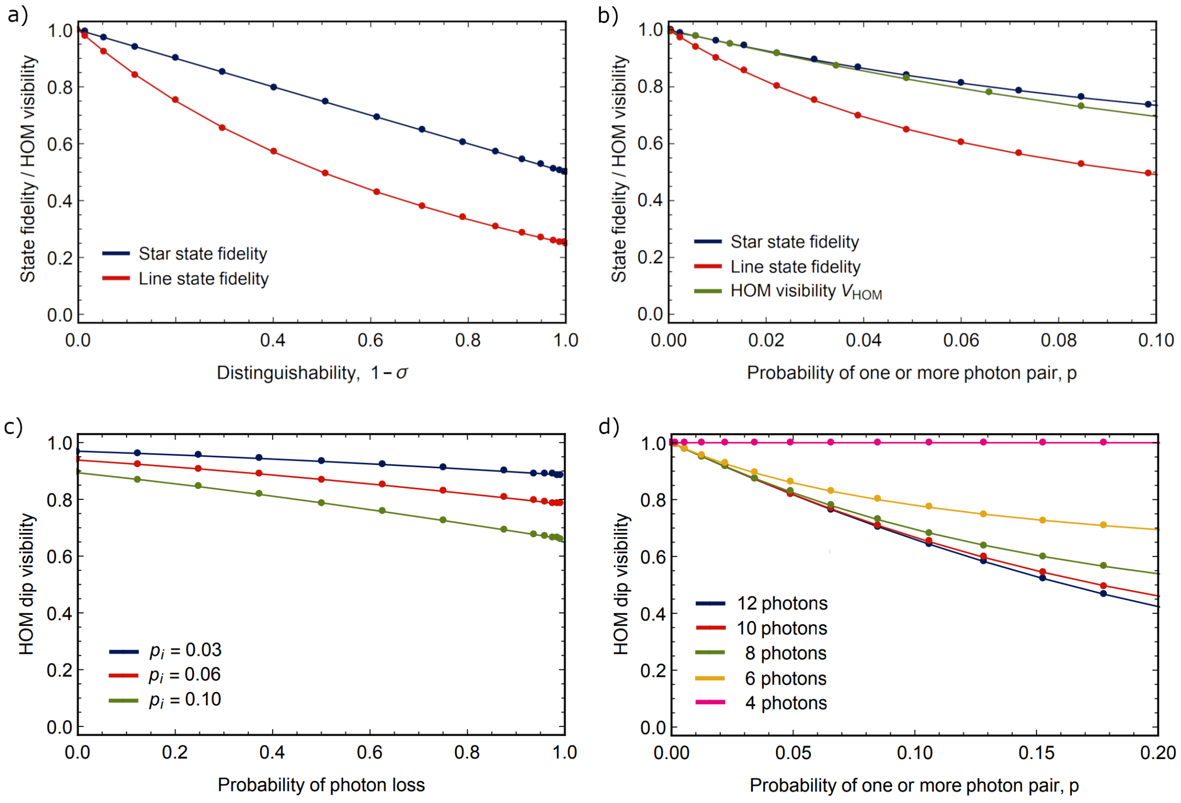

Supplementary Figure 9: Simulating multiphoton emission and partial distinguishability. a) POMOS is used to find the effect of partial distinguishability on the fidelity of $|S_4\rangle$ and $|L_4\rangle$. HOM dip visibility follows the same curve as the star graph state fidelity. b) PERM is used to compute the effect of multiphoton emission. Here, detections of up to eight photons are computed with a detection probability of -19 dB ≈ 0.01. c) The effect of multiphoton noise with varying amounts of loss on HOM-dip visibility, calculated with PERM. Here, up to ten photons are simulated. d) HOM dip visibility for increasing numbers of photon pairs in PERM. Truncating the calculation at eight photons is a good approximation for $p < 0.06$.

$\mathcal{M}^S_{II}$	$\lvert\langle\mathcal{M}^S_{II}\rangle\rvert$	$\mathcal{M}^L_{II}$	$\lvert\langle\mathcal{M}^L_{II}\rangle\rvert$
$g_4+g_{234}+g_{124}+g_{134}$	3.06 ± 0.06	$g_1+g_{12}+g_{13}+g_{123}$	2.5 ± 0.2
$g_{24}+g_{34}+g_{14}+g_{1234}$	3.14 ± 0.06	$g_1+g_{13}+g_{124}+g_{1234}$	2.6 ± 0.1
$g_4+g_{14}+g_{24}+g_{124}$	3.16 ± 0.04	$g_{12}+g_{14}+g_{123}+g_{134}$	2.5 ± 0.1
$g_4+g_{24}+g_{34}+g_{234}$	3.14 ± 0.05	$g_{14}+g_{124}+g_{134}+g_{1234}$	2.6 ± 0.1
$g_4+g_{14}+g_{34}+g_{134}$	3.03 ± 0.05	$g_{12}+g_{23}+g_{124}+g_{234}$	2.6 ± 0.1
$g_{34}+g_{234}+g_{134}+g_{1234}$	3.04 ± 0.07	$g_{23}+g_{34}+g_{134}+g_{123}$	2.6 ± 0.2
$g_{14}+g_{124}+g_{134}+g_{1234}$	3.06 ± 0.06	$g_{14}+g_{34}+g_{124}+g_{234}$	2.6 ± 0.1
$g_{24}+g_{124}+g_{234}+g_{1234}$	3.17 ± 0.06		

Supplementary Table 1: Result of every two-setting Mermin test that can be generated from the stabilisers of the star and line graph states. $\mathcal{M}^S_{II'}$ and $\mathcal{M}^L_{II'}$ are separated by a gap.

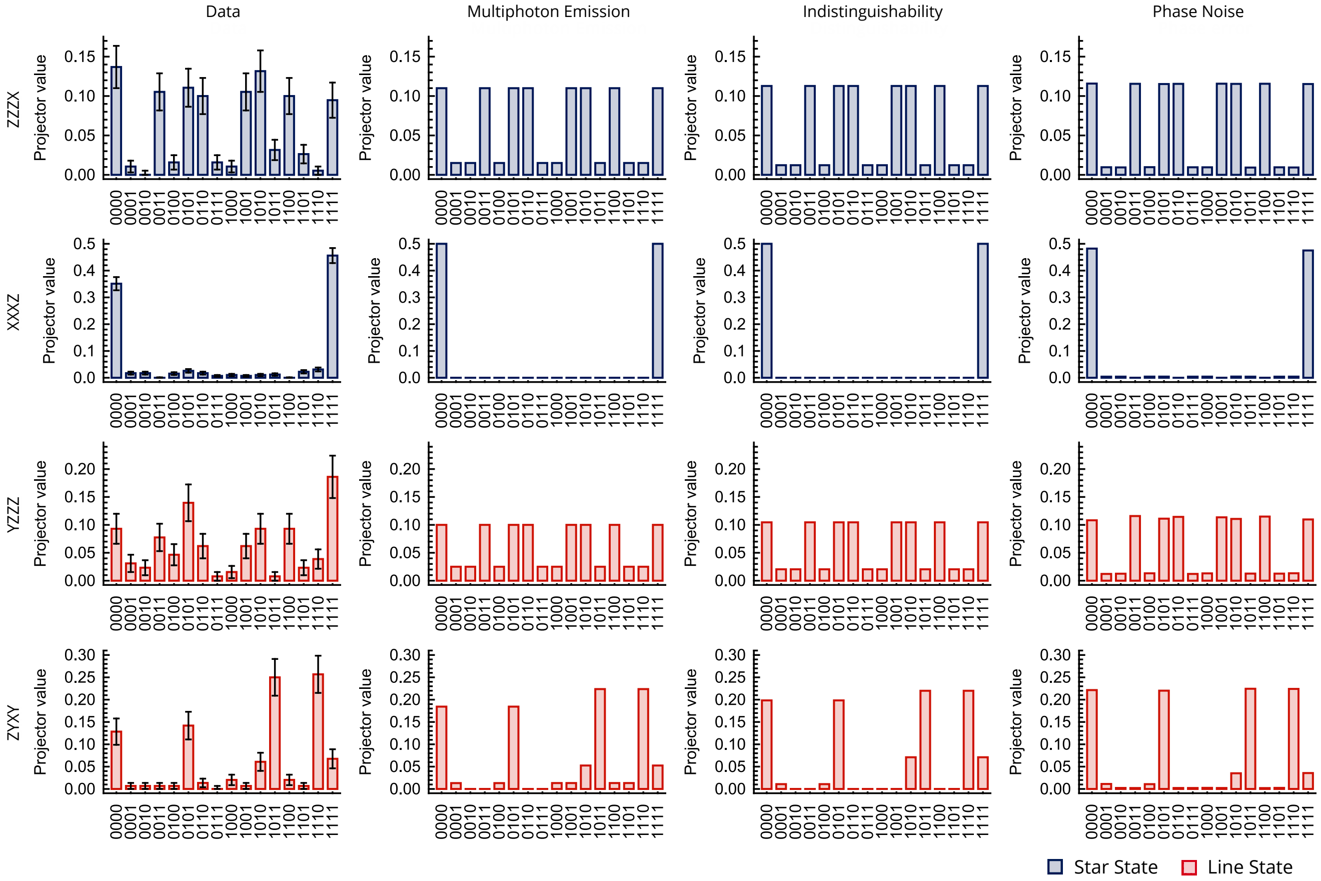

Supplementary Figure 10: Fitting model to data with Bayesian parameter estimation. Measured stabiliser data is shown in the left column for stabilisers ZZZX, XXXZ, YYZZ and ZYXY. Modelling each noise source independently, we fit the model for the each type of noise to the data using Bayesian parameter estimation. Columns 2, 3 and 4 show the simulated data that is best fit to the measured data, according to our Bayesian parameter estimate, for multiphoton emission, indistinguishability and phase noise respectively. Error bars on measured data represent the standard error, assuming a Poissonian distribution of the measured counts.

Supplementary References

[1] Silverstone, J. W., Bonneau, D, Ohira, K, Suzuki, N, Yoshida, H, Iizuka, N, Ezaki, M, Natarajan, C. M., Tanner, M. G., Hadfield, R. H., Zwiller, V, Marshall, G. D., Rarity, J. G., O'Brien, J. L. & Thompson, M. G. On-chip quantum interference between silicon photon-pair sources. *Nature Photonics* **8**, 104–108 (2014).

[2] Rarity, J. G., Ridley, K. D. & Tapster, P. Absolute measurement of detector quantum efficiency using parametric downconversion. *Applied Optics* **26**, 4616–4619 (1987).

[3] Christ, A., Laiho, K., Eckstein, A., Cassemiro, K. N. & Silberhorn, C. Probing multimode squeezing with correlation functions. *New Journal of Physics* **13**, 033027 (2011).

[4] Rarity, J. G., Tapster, J. R., Jakeman, E., Larchuk, T., Campos, R. A., Teich, T. C. & Saleh, B. E. A. Two-photon interference in a mach-zehnder interferometer. *Physical review letters* **65**, 1348 (1990).

[5] Faruque, I. I., Sinclair, G. F., Bonneau, D., Rarity, J. G. & Thompson, M. G. On-chip quantum interference with heralded photons from two independent micro-ring resonator sources in silicon photonics. *Optics Express* **26**, 20379–20395 (2018).

[6] Ding, Y., Peucheret, C., Ou, H. & Yvind, K. Fully etched apodized grating coupler on the soi platform with- 0.58 db coupling efficiency. *Optics letters* **39**, 5348–5350 (2014).

[7] Dumais, P., Wei, Y., Li, M., Zhao, F., Tu, X., Jiang, J., Celo, D., Goodwill, D., Fu, H., Geng, D. & Bernier, E. 2x2 multimode interference coupler with low loss using 248 nm photolithography. In *Optical Fiber Communication Conference*, W2A–19 (2016).

[8] Bellegarde, C., Pargon, E., Sciancalepore, C., Petit-Etienne, C., Hugues, V., Robin-Brosse, D., Hartmann, J & Lyan, P. Improvement of sidewall roughness of submicron soi waveguides by hydrogen plasma and annealing. In *2018 IEEE Photonics Conference (IPC)*, 1–4 (2018).

[9] Anant, V. Photon Spot. https://www.photonspot.com/ (2019).

[10] Ansari, V., Harder, G., Allgaier, M., Brecht, B. & Silberhorn, C. Temporal-mode measurement tomography of a quantum pulse gate. *Physical Review A* **96**, 063817 (2017).

[11] Aaronson, S. & Arkhipov, A. The computational complexity of linear optics. In *Proceedings of the forty-third annual ACM symposium on Theory of computing*, 333–342 (2011).
